# Retraction note: Histopathological features of bone regeneration in a canine segmental ulnar defect model

**DOI:** 10.1186/s13000-016-0573-4

**Published:** 2016-11-02

**Authors:** Rahim Hobbenaghi, Pariya Mahboob, Siamak Saifzadeh, Javad Javanbakht, Javad Yaghoobi Yeganeh Manesh, Rasool Mortezaee, Seyed Rashid Touni, Ehsan Hosseini, Shahin Aghajanshakeri, Milad Moloudizargari, Soheil Javaherypour

**Affiliations:** 1 Department of Pathology, Faculty of Veterinary Medicine, Urmia University, Urmia, Iran; 2 Graduate, Faculty of Veterinary Medicine, Urmia University, Urmia, Iran; 3 Institute of Health and Biomedical Innovation, Queensland University of Technology, 60 Musk Avenue, Kelvin Grove, Brisbane, QLD 4059 Australia; 4 Department of Pathobiology, Faculty of Veterinary Medicine, Tehran University, Tehran, Iran; 5 Graduate of Islamic Azad University of Shahrekord, Faculty of Veterinary Medicine, Shahrekord University, Shahrekord, Iran; 6 Student of Ferdowsi University of Mashhad, Faculty of Veterinary Medicine, Ferdowsi University, Mashahd, Iran; 7 Ph.D Student of Anatomy and Embryology, Faculty of Veterinary Medicine, Urmia University, Urmia, Iran; 8 Faculty of Para Veterinary Medicine, Ilam University, Ilam, Iran; 9 Student of Veterinary Medicine, Faculty of Veterinary Medicine, Urmia University, Urmia, Iran

The Editor-in-Chief and Publisher have retracted this article [1] because the scientific integrity of the content cannot be guaranteed. An investigation by the Publisher found it to be one of a group of articles we have identified as showing evidence suggestive of attempts to subvert the peer review and publication system to inappropriately obtain or allocate authorship. This article showed evidence of plagiarism (most notably from the articles cited [2–5]) and peer review and authorship manipulation.
